# *Drosophila Eyes Absent* Is Required for Normal Cone and Pigment Cell Development

**DOI:** 10.1371/journal.pone.0102143

**Published:** 2014-07-24

**Authors:** Umesh C. Karandikar, Meng Jin, Barbara Jusiak, SuJin Kwak, Rui Chen, Graeme Mardon

**Affiliations:** 1 Department of Pathology and Immunology, Baylor College of Medicine, Houston, Texas, United States of America; 2 Program in Developmental Biology, Baylor College of Medicine, Houston, Texas, United States of America; 3 Department of Molecular and Human Genetics, Baylor College of Medicine, Houston, Texas, United States of America; 4 Human Genome Sequencing Center, Baylor College of Medicine, Houston, Texas, United States of America; 5 Department of Neuroscience, Baylor College of Medicine, Houston, Texas, United States of America; 6 Department of Ophthalmology, Baylor College of Medicine, Houston, Texas, United States of America; 7 Program in Cell and Molecular Biology, Baylor College of Medicine, Houston, Texas, United States of America; Indiana University, United States of America

## Abstract

In *Drosophila*, development of the compound eye is orchestrated by a network of highly conserved transcriptional regulators known as the retinal determination (RD) network. The retinal determination gene *eyes absent* (*eya*) is expressed in most cells within the developing eye field, from undifferentiated retinal progenitors to photoreceptor cells whose differentiation begins at the morphogenetic furrow (MF). Loss of *eya* expression leads to an early block in retinal development, making it impossible to study the role of *eya* expression during later steps of retinal differentiation. We have identified two new regulatory regions that control *eya* expression during retinal development. These two enhancers are necessary to maintain *eya* expression anterior to the MF (*eya-IAM*) and in photoreceptors (*eya-PSE*), respectively. We find that deleting these enhancers affects developmental events anterior to the MF as well as retinal differentiation posterior to the MF. In line with previous results, we find that reducing *eya* expression anterior to the MF affects several early steps during early retinal differentiation, including cell cycle arrest and expression of the proneural gene *ato*. Consistent with previous observations that suggest a role for *eya* in cell proliferation during early development we find that deletion of *eya-IAM* leads to a marked reduction in the size of the adult retinal field. On the other hand, deletion of *eya-PSE* leads to defects in cone and pigment cell development. In addition we find that *eya* expression is necessary to activate expression of the cone cell marker Cut and to regulate levels of the Hedgehog pathway effector Ci. In summary, our study uncovers novel aspects of *eya*-mediated regulation of eye development. The genetic tools generated in this study will allow for a detailed study of how the RD network regulates key steps in eye formation.

## Introduction

The early *Drosophila* eye imaginal disc consists of pluripotent cells that are committed to a retinal fate and express high levels of *eyeless* (*ey*), a critical regulator of eye development. These pluripotent retinal progenitors begin differentiation along a moving boundary called the morphogenetic furrow (MF) at the posterior margin of the early third instar eye imaginal disc [1], [2]. Several key cellular events take place immediately anterior to the advancing MF, including exit from the cell cycle and arrest in the G1 phase [3]. At the same time, retinal progenitors anterior to the MF initiate expression of the proneural gene *atonal* (*ato*), which encodes a basic helix-loop-helix (bHLH) transcription factor (TF) necessary for the onset of photoreceptor differentiation [4]. Posterior to the MF, *ato* expression resolves into regularly spaced single cells, which differentiate into R8 photoreceptors, the founding cell of each ommatidium (unit eye). The remaining cell types of the adult eye are progressively recruited and differentiate around each founding R8 photoreceptor [5].

The RD network member *eyes absent* (*eya*) is a conserved transcription co-factor expressed in a broad band anterior to the MF and in all cells posterior to the MF [6] Mutations in *eya* such as *eya^1^* and *eya^2^* lead to eye specific loss of *eya* expression and gives rise to adult flies without eyes, suggesting that *eya* is necessary for eye development [7], [8]. *eya^1^* and *eya^2^* imaginal discs express *ey* suggesting that the retinal progenitors lacking *eya* expression are committed to the eye lineage [7]. However, cells lacking *eya* expression do not execute any further steps of eye development [7], [8], [9], including G1 arrest, MF initiation, or *ato* expression [10]. In clones of *eya* mutant tissue, retinal progenitors overproliferate and grow out of the plane of the eye disc; these cells eventually undergo apoptosis [10]. Due to the early blocks in retinal differentiation caused by loss of *eya* expression, the role *eya* may play in differentiating photoreceptors has been difficult to study. One way to bypass this early block and to identify the role of *eya* during later steps in retinal developmental is to generate single-celled *eya* mutant clones posterior to the MF. Although this approach has suggested a requirement for *eya* function in differentiating photoreceptors, it use is limited by the minimal clone size possible [10].

Previous studies suggest that Eya functions at least in part through forming a transcriptional regulatory complex with the homeodomain transcription factor Sine oculis (So), which directly activates expression of several regulators of retinal development [11], [12], [13], [14], [15]. Eya has been shown to physically interact with So [10], raising the possibility that Eya can regulate So targets during eye development. In line with this idea, forced expression of *eya* and *so* causes G1 arrest of proliferating retinal progenitors anterior to the MF [16]. Based on these observations it has been proposed that Eya plays an important role in several steps from initiation of the MF [9] and G1 arrest of retinal progenitors anterior to the MF [16], to terminal photoreceptor differentiation [10]. However, evidence for the role of *eya* during photoreceptor morphogenesis posterior to the MF is lacking. We have addressed this gap by the design and implementation of an *eya* genomic rescue construct that allows normal early (anterior) expression but blocks *eya* expression posterior to the MF, such that retinal differentiation is properly initiated but downstream developmental events are affected.

In this report we describe two new regulatory regions of *eya*, *eya-IAM* (**I**mmediately **A**nterior to the **M**F) and *eya-PSE* (**P**hotoreceptor **S**pecific **E**xpression). Reporter analysis of these regulatory regions using destabilized GFP (dGFP) suggests that *eya-IAM* is expressed in retinal progenitors immediately anterior to the MF, while *eya-PSE* is expressed in differentiating photoreceptors posterior to the MF in the third instar eye imaginal disc. We have analyzed the role of these elements in regulating *eya* expression during retinal development by deleting them in a previously characterized *eya* genomic rescue construct. As predicted, our analysis of *eya^GRΔIAM^* and *eya^GRΔPSE^* animals supports a role for *eya* during G1 arrest of retinal progenitors anterior to the MF as well as expression of the proneural gene *ato*. Our analysis also suggests a novel role for *eya* in the development of cone and pigment cells.

## Materials and Methods

### Construction of enhancer reporter lines

Enhancer fragments were amplified from a BAC carrying the *eya* genomic region. The enhancer fragments were then subcloned into a plasmid carrying destabilized GFP (dGFP) as a reporter and an *attB* site for site-specific integration [17]. The *eya-IAM* fragment was generated using IAM-F 5′ AGAGAATTCCAAACACCTGGCATTATCGCTTCATCTCGG and IAM-R 5′AGCGGATCCAGTTTCGTCTCCTCTTTTGCTGCCTCTTTG, the *eya-PSE* enhancer was generated using PSE-F 5'-ATTGAATTCGTCCAGAGTGGTGGTGGTGA and PSE R 5'-GATCTCGAGGTACGATTTGTGCGTGCG. These primers also indicate the limits of deletion. The resulting construct was integrated into the *P{CaryP}attP2* site (abbreviated *P2*), which is located at 3L∶11,063,638, using φC31 integrase. Site-specific integration was confirmed by PCR with *attB/attP* primers [18].

### Recombineering-induced deletions of enhancers in the *eya* genomic rescue

The *eya* genomic rescue (*eya^GR^*) constructs carrying deletions of *eya-IAM* and *eya*-*PSE* enhancers were generated using recombineering as previously described [18]. The recombineered deletion transgenes were injected into *P2*, the same site used for the wild-type *eya^GR^* [19], and site-specific integration was verified by PCR with *attB/attP* primers. We performed PCR on genomic DNA from the transgenic flies to verify the deletions.

### *Drosophila* genotypes

All crosses were performed on standard cornmeal agar at 25°C. Heat shocks were performed at 37°C as described in Anderson et.al., 2012. To test the function of the *eya* enhancers in eye development, we crossed the mutant *eya^GR^* constructs into the following mutant backgrounds: *eya^2^* homozygotes, which specifically lack Eya expression in the eye disc [7], and *eya^cliIID^/Df(2L)BSC354. eya^cliIID^* is a null allele caused by a premature stop codon that causes recessive embryonic lethality [20], and *Df(2L)BSC354* (hereafter referred to as *Df(eya)* removes the entire *eya* locus [21]. Consequently, *eya^cliIID^/Df (eya)* lacks all endogenous *eya* function. The *eya^ΔIAM^* and *eya^ΔPSE^* ^clones^ were generated by crossing *eya^cliIID^, FRT 40/CyO; eya^GRΔIAM^/TM6B,Tb^1^* or *eya^cliIID^, FRT 40/CyO; eya^GRΔPSE^/TM6B,Tb^1^* respectively with *hsflp*; *P{w[+mC] = arm-LacZ}, FRT40/CyO* animals. The RNAi stocks *w; UAS-soRNAi* (transformant ID104386) and *w; UAS-eyaRNAi* (transformant ID 108071) from VDRC were used [17], [22].

### Immunohistochemistry of third instar eye discs, pupal eye discs, and imaging of adult eyes and plastic sections

Primary antibodies used were mouse anti-Eya (1∶200), anti-Cyclin B (1∶200), Anti-Ci (1∶200), anti-Cut(1∶100), rat anti-Elav (1∶500) (Developmental Studies Hybridoma Bank), guinea pig anti-Sens (1∶2000), guinea pig anti-Ato (1∶1000, gift from H. Bellen), Rabbit anti-β-Gal (1∶1000, Promega) and chick anti-GFP (1∶1000, Abcam). All secondary antibodies were made in goat and used at a final dilution of 1∶500; Cy3 and Cy5 labeled secondary antibodies were obtained from Jackson ImmunoResearch, the Alexa Fluor 488 (used at 1∶500) and Phalloidin Alexa Fluor 594 (used at 1∶500) was obtained from life technologies. Images were taken with a Zeiss LSM 510 confocal microscope and processed with ImageJ and Adobe Photoshop software. Immunohistochemistry on third instar eye discs and imaging of the adult eye were conducted as described previously [17]. Staining of 48 hr pupal eye discs and the plastic sections of adult eyes were generated as previously described [23].

## Results

### Identification of distinct regulatory elements controlling retinal expression of *eya*

**Figure 1:**
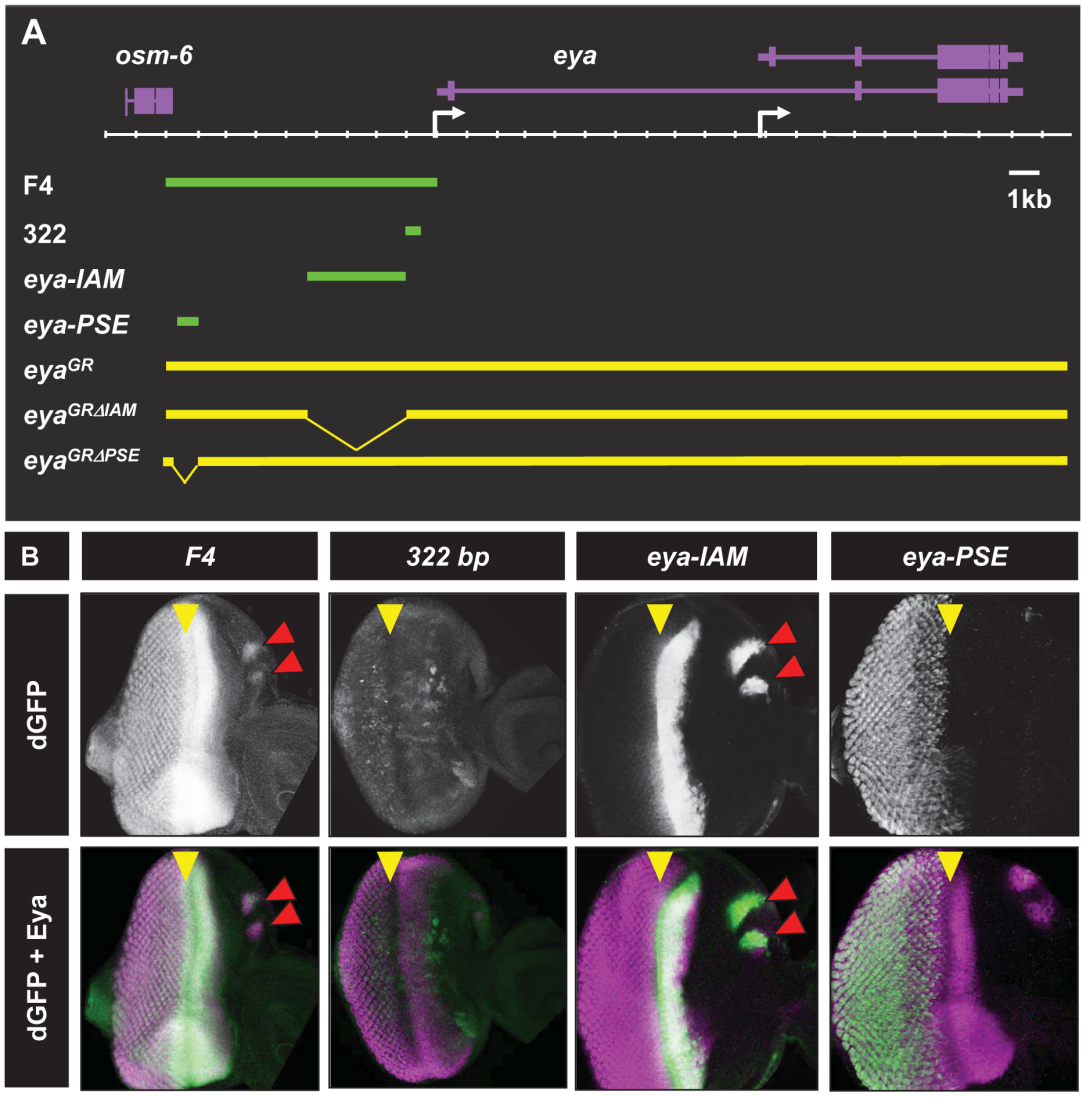
Identification of two new *cis* retinal enhancers of *eya*. A. Schematic of the *eya* locus showing enhancer fragments and genomic constructs. Genes (purple) are shown above the scale while the enhancer fragments (green bars) and genomic constructs (yellow) are on bottom. Transcription start sites are indicated by white arrows. B. Third instar eye discs showing enhancer-driven dGFP expression (white in top panels and green in bottom panels) along with endogenous Eya expression (magenta in bottom panels). Yellow arrowheads indicate the position of the MF and the red arrowheads mark ocellar dGFP expression in *eya-IAM* animals.

The eye-specific *eya* mutant *eya^2^* harbors a 322 bp deletion approximately 500 bp upstream of the *eya* transcription start site [24]. This 322 bp deletion leads to a complete loss of *eya* expression in the eye imaginal disc, suggesting a necessary role for this region for *eya* expression during eye development. A previous report using LacZ reporter constructs suggests that the 322 bp region can recapitulate the basic pattern of *eya* expression anterior to the MF in the early larval eye disc [25]. However, the prolonged stability of LacZ and the low resolution of the activity staining method used in this study precluded a detailed understand of the transcriptional activity of the 322 bp segment. To further refine the transcriptional limits of this DNA, we tested the ability of this 322 bp region to drive destabilized GFP (dGFP) as a reporter. Due to its short half-life, dGFP has proven to be a far more accurate reporter of transcriptional activity than either eGFP of LacZ ([17], [26] and R.C. unpublished results). Consistent with previous reports, we find that the 322 bp enhancer is sufficient to drive early dGFP expression [25]. In contrast, however, our analysis suggests that 322 bp-driven dGFP expression in the early third instar is irregular (Figure S1A) by the early third instar stage, reporter expression begins to weaken (Figure S1C), and by mid to late third instar stage 322 bp driven dGFP expression is completely lost (Figure 1B). These results suggest that the 322 bp enhancer does not recapitulate the complete endogenous *eya* expression pattern during retinal development. Therefore, to identify the regulatory elements needed for complete retinal expression of *eya* we generated a large reporter construct, *F4-dGFP* that contains ∼9 kb of DNA upstream of the *eya* transcriptional start site (TSS), including the previously identified 322 bp enhancer (Figure 1A). We find that *F4-dGFP* recapitulates the complete endogenous *eya* expression pattern in the developing eye disc (Figure 1B and Figure S2A-A‴). In the second instar eye disc, *F4*-*dGFP* expression appears as a posterior to anterior gradient mimicking the endogenous *eya* gradient (data not shown). Posterior to the MF in third instar eye disc, F4 drives dGFP expression in differentiating photoreceptors and still maintains dGFP expression in retinal progenitors anterior to the MF (Figure 1B). Therefore, the ∼9 kb region upstream of the distal *eya* promoter harbors regulatory elements that are sufficient to recapitulate the endogenous *eya* expression in the developing eye imaginal disc.

### *eya-IAM* and *eya-PSE* enhancers are active during different stages of retinal development

The *F4* region includes the 322 bp enhancer previously shown to be necessary for *eya* expression during eye development [24], [25]. To identify additional regulatory elements that are sufficient for complete retinal expression of *eya*, we analyzed different sub-fragments of *F4*. Through this analysis we have identified two separate regions that are named *eya-IAM* (**I**mmediately **A**nterior to the **M**F) and *eya-PSE* (**P**hotoreceptor **S**pecific **E**nhancer), based on their ability to drive dGFP expression in undifferentiated and differentiating retinal cells, respectively (Figure 1A,B).

*eya-IAM* is a ∼3 kb fragment immediately upstream of the previously identified 322 bp enhancer (Figure 1A). *eya-IAM* expression begins as diffuse band at the posterior margin of the eye disc at ∼68 hr after egg laying (AEL, Figure S1B). After MF initiation, *eya-IAM* driven dGFP expression becomes more intense and is maintained at high levels anterior to the MF (Figure 1B). Both *F4* as well as *eya-IAM* fragments drive the dGFP reporter expression that overlaps the broad band of endogenous *eya* expression immediately anterior to the MF (Figure 1B). These observations suggest that *eya-IAM* is sufficient for *eya* expression anterior to the MF.

The 785 bp *eya-PSE* fragment drives dGFP expression posterior to the MF in differentiating photoreceptors (Figure1B). *eya-PSE* driven dGFP expression is completely absent from second instar and early third larval instar eye discs (data not shown). *eya-PSE* driven dGFP co-localizes with Eya staining in photoreceptors posterior to the MF (Figure 1B). Co-staining with the neuronal differentiation marker Elav suggests that *eya-PSE* drives expression in the differentiating neurons (Figure 1B and Figure S3). *eya-PSE* driven dGFP expression is absent from undifferentiated cells posterior to the MF. Thus, *eya-IAM* and *eya-PSE* are sufficient to drive reporter gene expression closely mimicking the endogenous *eya* pattern, raising the possibility that they may also be required for proper *eya* expression during normal eye development.

### *eya-IAM* is required for normal retinal development

**Figure 2:**
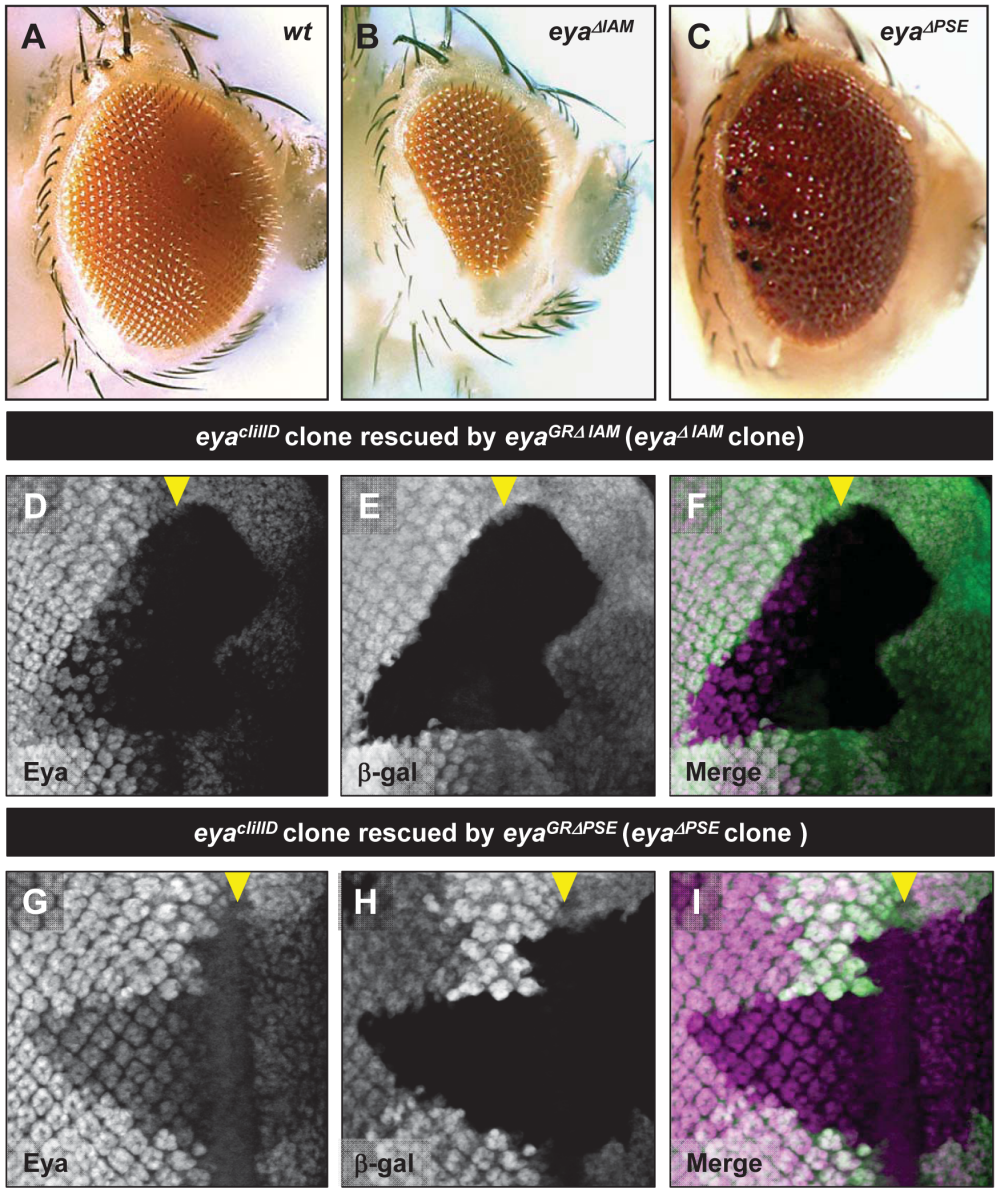
The *IAM* and *PSE* enhancers are required for *eya* expression during normal retinal development. A-C. Adult eye phenotype of wild-type (A), *eya^cliIID^/Df* (*eya*); *eya^GRΔIAM^* (B), *eya^cliIID^/Df* (*eya*); *eya^GRΔPSE^* (rare escaper, C), D-I Clones of *eya^cliIID^* rescued by *eya^GRΔIAM^* harboring a deletion of *eya-IAM* (D-F) and rescued by *eya^GRΔPSE^* harboring a deletion of *eya-PSE* (G-I). The *eya^cliIID^* clones are marked by the absence β-galactosidase expression (white in E and H and green in I and J) showing reduced Eya expression (white in D and G and magenta in F and I). Yellow arrowheads indicate the position of the MF.

We tested if the *eya-IAM* enhancer is required for *eya* expression during normal eye development by deleting this region from a previously reported *eya* genomic rescue, *eya^GR^*, that is sufficient to fully rescue all known *eya* mutant phenotypes (henceforth referred to as *eya^GR^*, [19], [27]. The genomic rescue harboring the deletion of *eya-IAM* (*eya^GRΔIAM^*, Figure 1A) rescues the lethality of *eya^cliIID^/Df* animals, however these rescued animals have a markedly reduced retinal field as compared to controls and wild type animals (Figure 2A, B and Figure S1H). Additionally, consistent with the ability of *eya-IAM* to drive expression of dGFP in ocelli (Figure 1B, red arrows) deletion of *eya-IAM* also leads to complete loss of ocelli in *eya^cliIID^/Df* animals (Figure S1D, E). The ability of *eya^GRΔIAM^* to rescue the lethality of the *eya^cliIID^* allele suggests that *eya-IAM* may not play a major role in regulating the expression of *eya* during embryogenesis. Additionally, *eya^GRΔIAM^*, partially rescues the complete loss of the retinal field observed in the eye specific *eya* allele *eya^2^/Df(eya)* animals (Figure S1J).

### *eya-IAM* is required for normal *eya* expression anterior to the MF

**Figure 3:**
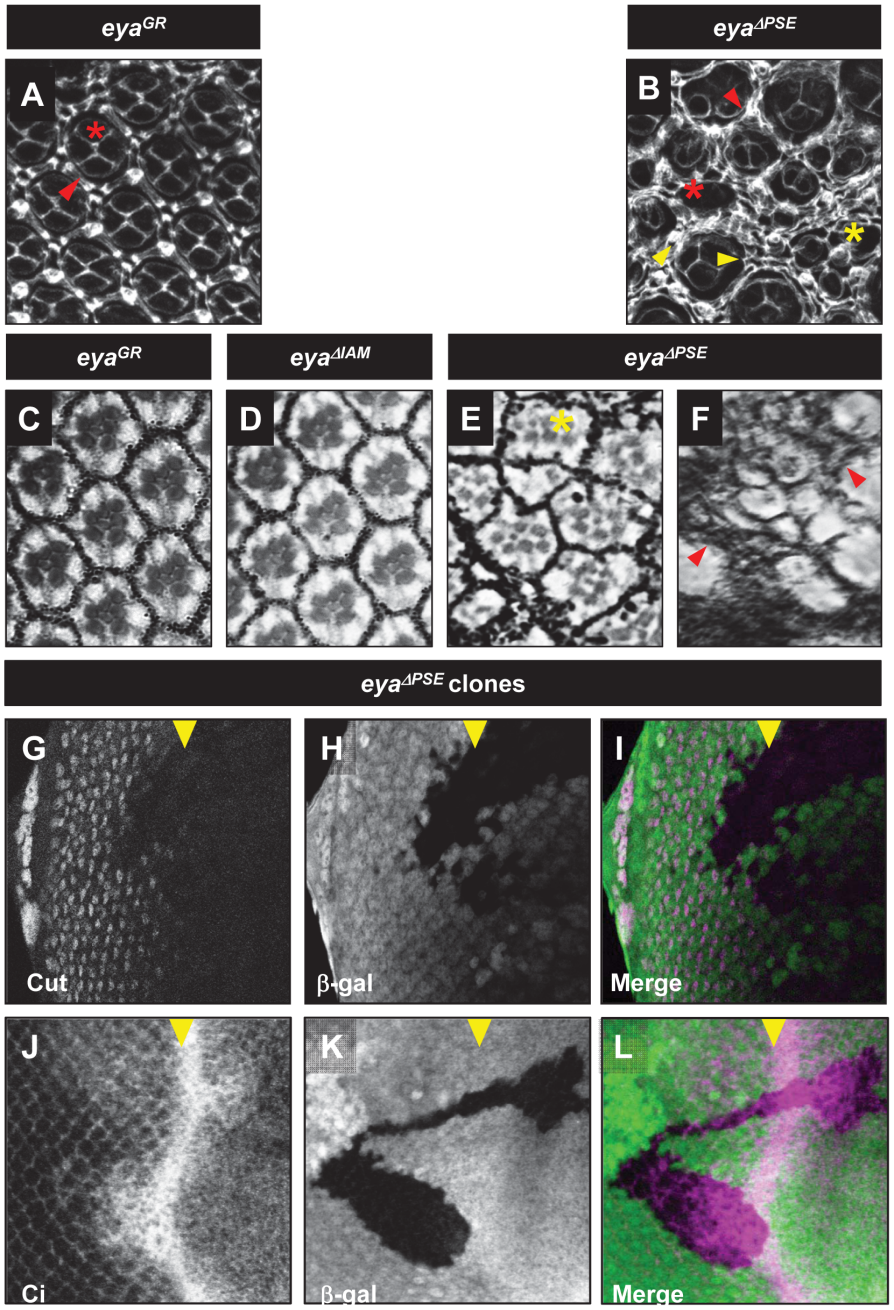
*eya* is required for development of cone and pigment cells. Pupal retina (A, B), Adult plastic sections (C-F) of *eya^GR^* and *eya^ΔPSE^*. Clones of *eya^ΔPSE^* in third instar eye disc (G-L). Pupae stained for Dlg (white, A and B) show cone cells and pigment cells. The adult eye sections show the rhabdomere structure of adult eyes. *eya^GR^* (A and C) *eya^GRΔPSE^* (B, E and F) *eya^GRΔIAM^* (D). Arrow heads indicate pigment cells and asterisks show cone cells, respectively. Clones of *eya^cliIID^* rescued by *eya^GRΔPSE^* stained for Cut (white in G, magenta in I) and Ci expression (white in J, magenta in L). *eya^cliIID^* clones are marked by the absence of β-galactosidase expression (white in H and K, green in I and L).

Since *eya-IAM* is sufficient to drive strong reporter expression immediately anterior to the MF in mid-third instar eye discs, we tested if *eya-IAM* is required for normal Eya expression *in vivo*. This was analyzed in the eye discs of *eya^cliIID^/Df; eya^GRΔIAM^/+* animals (henceforth referred to as *eya^ΔIAM^*), as well as *eya^cliIID^* clones rescued by a single copy of *eya^GRΔIAM^* (henceforth referred to as *eya^ΔIAM^* clones). As expected (assuming there are no other redundant enhancers), Eya expression immediately anterior to the MF in *eya^ΔIAM^* eye discs is significantly lower compared to control animals (Figure S1F,G). Moreover, *eya^ΔIAM^* clones show a complete loss of Eya expression anterior to the MF as compared to neighboring control tissue (Figure 2D-F). Both observations were verified in multiple *eya^ΔIAM^* eye discs and in *eya^ΔIAM^* anterior clones that do not span the MF (data not shown). Thus, consistent with the *eya-IAM-dGFP* reporter expression pattern, deletion of *eya-IAM* results in reduced *eya* expression in retinal progenitors anterior to the MF. In addition, *eya^ΔIAM^* clones posterior to the MF also show a reduction in the levels of Eya (Figure 2D-F), suggesting that in a genomic context *eya-IAM* may be required to maintain *eya* expression throughout retinal development. However the *eya^ΔIAM^* clones in the adult eye appear indistinguishable from heterozygous tissue. Therefore it is possible that loss of *eya* expression due to deletion of *eya-IAM* may not affect normal retinal development posterior to the MF. Consistent with this interpretation, sections of adult *eya^ΔIAM^* eyes show the normal complement of rhabdomeres (compare Figure 3C and D), suggesting that deletion of *eya-IAM* does not affect specification or differentiation of retinal cells posterior to the MF.

### *eya-PSE* is required for normal retinal development

The *eya-PSE* enhancer is sufficient to drive dGFP expression in differentiating photoreceptors posterior to the MF. Therefore, we tested if *eya-PSE* is required for normal Eya expression posterior to the MF. We generated *eya^GRΔ^* ^PSE^, an *eya^GR^* construct that harbors a deletion of *eya-PSE* (Figure 1A) and tested its ability to rescue retinal development in the absence of endogenous *eya* function. *eya^GRΔPSE^* is largely unable to rescue the lethality of *eya^cliIID^*/Df animals, suggesting a role for the *eya-PSE* enhancer in additional tissues during development. However, rare *eya^cliIID^/Df; eya^GRΔPSE^/+* (henceforth referred to as *eya^ΔPSE^*) escapers do reach adulthood and eclose. Such *eya^ΔPSE^* escapers are unable to locomote normally and rapidly fall into the fly food and die. However, *eya^ΔPSE^* escapers do show strong eye defects, including a glazed appearance and occasional melanotic tissue on the eye surface. These defects are more severe toward the posterior margin of the eye as compared to the anterior region (Figure 2C). Consistent with this result, adult eyes of *eya^ΔPSE^* escapers show disorganized ommatidia in the anterior region (Figure 3E) while the posterior region shows a drastic loss of rhabdomeres and an excess of pigment cells (Figure 3F).

### *eya-PSE* is required for normal retinal *eya* expression

**Figure 4:**
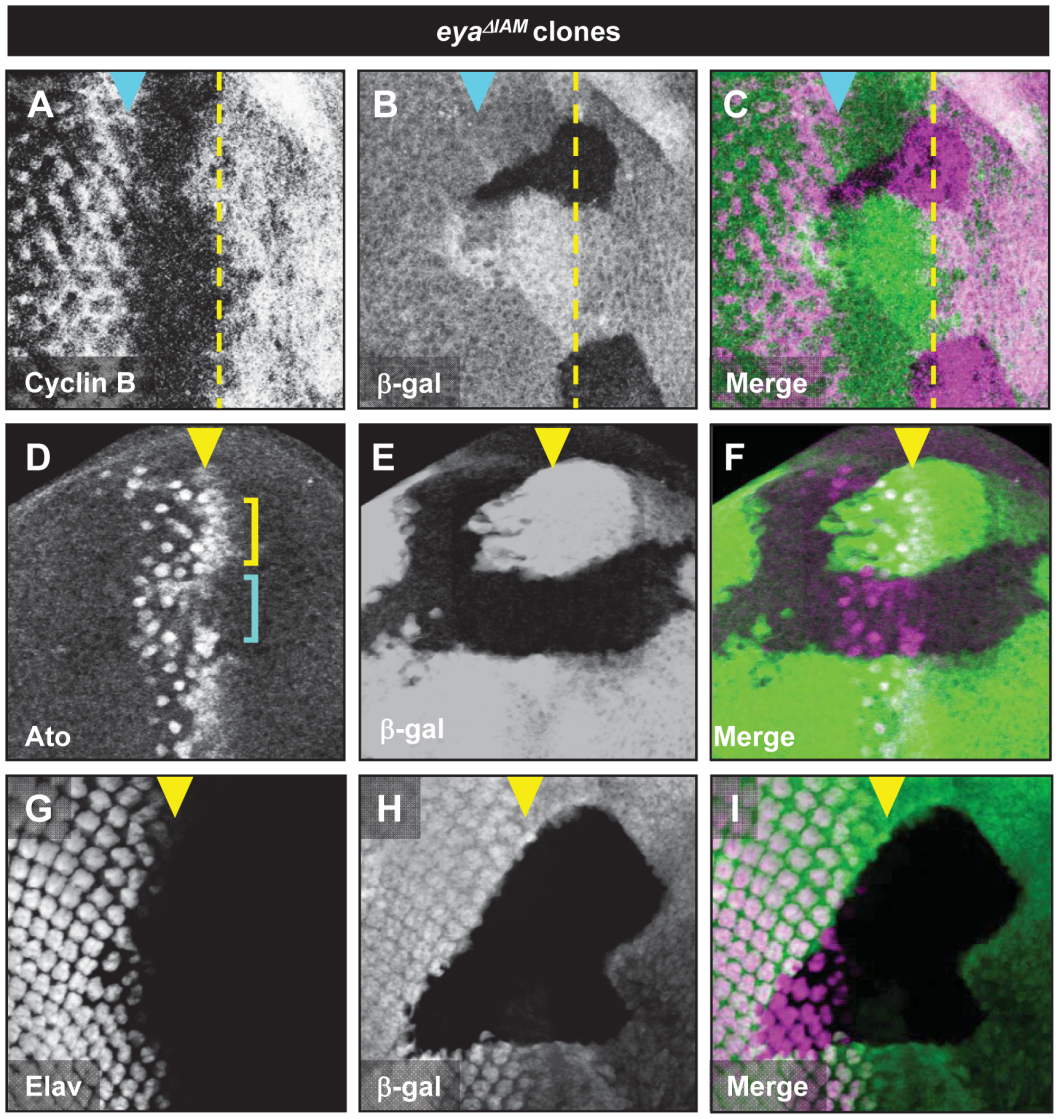
*eya* is required for normal Cyclin B and Ato expression. Cyclin B (A), Ato (B) and Elav (C) expression in *eya^cliIID^* clones rescued by *eya^GRΔIAM^*. *eya^cliIID^* clones are marked by the absence of β-galactosidase expression (green in B, C, E, F, H, and I). Yellow dashed lines and arrowheads indicate the position of the MF, cyan arrowheads in A-C indicate the position of the second mitotic wave. In panel D, cyan and yellow brackets mark Ato expression in *eya^cliIID^* clones and *eya^cliIID^* heterozygous tissue, respectively.

To test if the *eya-PSE* deletion affects Eya expression posterior to the MF, we analyzed *eya^ΔPSE^* clones (*eya^cliIID^* clones rescued by a single copy of *eya^GRΔPSE^*). In contrast to *eya^ΔPSE^* whole mutant escaper animals, *eya^ΔPSE^* clones show a clear reduction in the levels of Eya expression posterior to the MF (Figure 2G-I). *eya^ΔPSE^* clones that do not overlap the MF also show a severe reduction of *eya* expression (data not shown). As seen in Figure 2G-I, *eya^ΔPSE^* clones anterior to the MF also show a reduction in the levels of Eya expression, although not as severe as that seen in *eya^ΔIAM^*clones (compare Figure 2D and G). These observations have been verified in at least 5 independent clones for each genotype. The images in the figure 2D-I were captured and processed at identical settings to enable a direct comparison. Interestingly, while the *eya-PSE* enhancer is not sufficient to drive reporter expression in retinal cells anterior to the MF, *eya^ΔPSE^* clones anterior to the MF show a marked reduction in the levels of Eya (Figure 2D-F), suggesting that in the genomic context, *eya-PSE* may be required for normal Eya expression anterior to the MF. However, expression of Elav, a marker of photoreceptor differentiation, in *eya^ΔPSE^* clones is not significantly affected (Figure S2B) compared to *eya^ΔIAM^* clones (Figure 4G-I), suggesting that the reduction of *eya* expression anterior to the MF due to deletion of *eya-PSE* may not be sufficient to delay retinal specification and recruitment of photoreceptors.

### Normal Eya expression anterior to the MF is required for G1 arrest and initiation of retinal differentiation

Signals emanating from the MF play a critical role in the developmental events that take place anterior to the MF, such as mediating G1 arrest [28], [29], [30], [31] and induction of *ato* expression [29]. Loss of *eya* leads to a block of MF initiation and progression, precluding a detailed study of the role *eya* plays in the developmental events associated with MF movement. Since deletion of *eya-IAM* leads to a reduction of Eya and yet still allows initiation of retinal differentiation, we analyzed the effects of reducing *eya* expression anterior to the MF on both G1-arrest and *ato* induction. Specifically, we used *eya^ΔIAM^* clones to create retinal progenitors with reduced *eya* expression anterior to the MF.

G1 arrest was analyzed by monitoring Cyclin B expression. Normally, Cyclin B is absent from G1 arrested cells and is expressed in cells in the G2 and M phases [32]. We find that reduced levels of *eya* expression in *eya^ΔIAM^* clones leads to elevated Cyclin B expression in retinal progenitors that would normally undergo G1 arrest (Figure 4A-C). These results suggest that retinal progenitors with reduced levels of *eya* expression may not undergo efficient G1 arrest anterior to the MF. *eya^ΔIAM^* clones anterior to the MF also show decreased levels of *ato* expression in the pre-proneural zone (Figure 4D, compare the Ato expression indicated by cyan and the yellow brackets). While the defects in the Cyclin B and Ato expression due to reduced levels of *eya* are resolved as the MF progresses, these observations suggest that retinal progenitors with reduced levels of *eya* show a delay in developmental events prior to photoreceptor differentiation. Consistent with these observations, *eya^ΔIAM^* clones that span the MF also show a slight delay in the onset of photoreceptor differentiation (Figure 4G-I). Taken together, these observations suggest that *eya* expression is required for events associated with MF movement, both anterior and posterior to the MF.

### *eya* expression is required for cone cell specification

**Figure 5:**
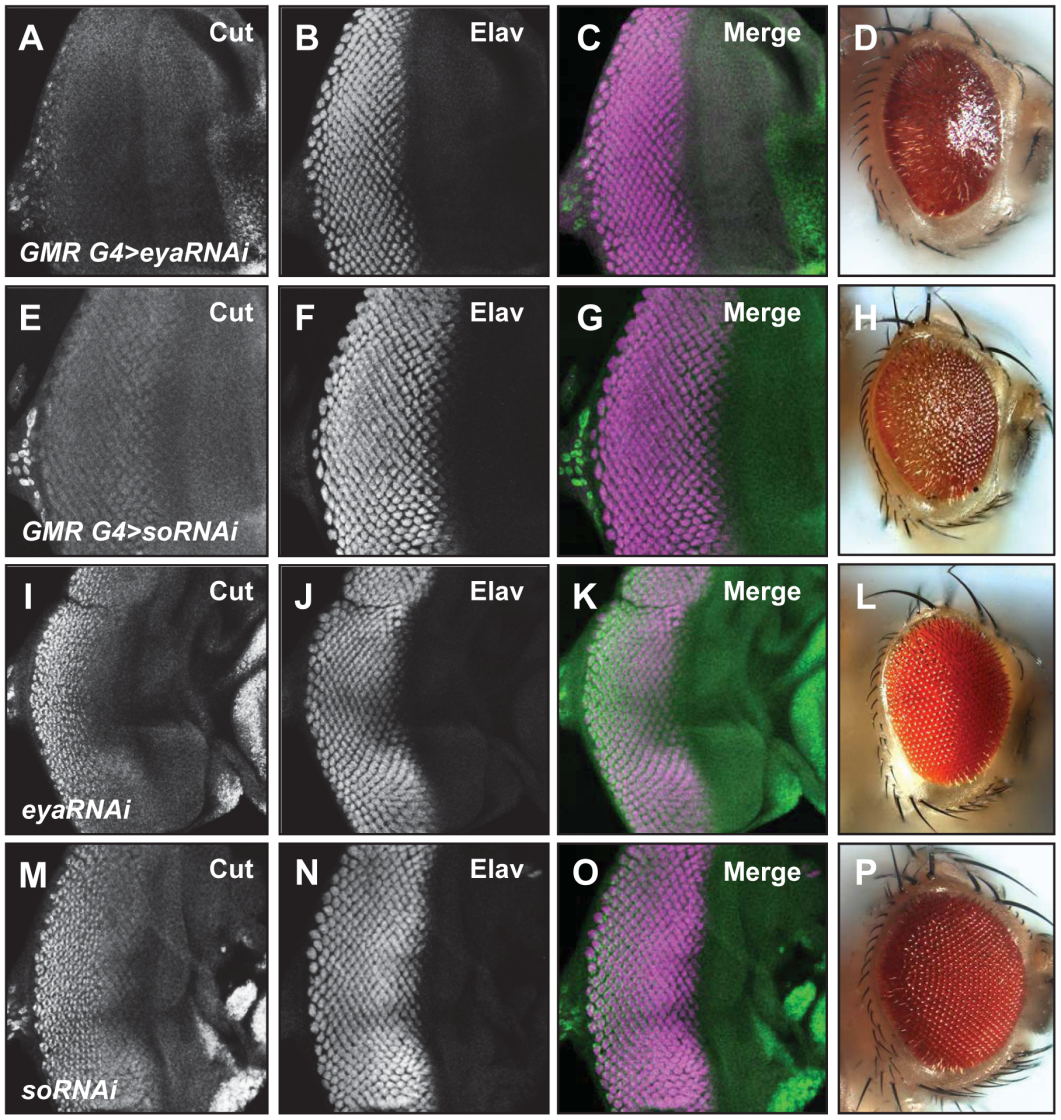
RNAi-mediated knockdown of *eya* or *so* strongly reduce Cut expression. Cut (green) and Elav (magenta) expression in third instar eye discs (A-C, E-G, I-K, and M-O) and adult eyes (D, H, L and P) are shown. *GMR-Gal4/+; UAS-eyaRNAi/+* (A-D), *GMR-Gal4; UAS-soRNAi* (E-H), *UAS-eyaRNAi/+* (I-L), *UAS-soRNAi*/+ (M-P).

Very little is known about the role of *eya* in the development of support cells within ommatidia. Therefore, we used *eya^ΔPSE^* clones to test if *eya* is required for cone and pigment cell development. We analyzed cone cell specification in *eya^ΔPSE^* clones using Cut as an early marker for cone cell fate [33]. We find that *eya^ΔPSE^* clones do not express Cut (Figure 3G-I). We further tested the role of *eya* and its interaction partner *so* in regulating cone cell specification by knocking down the expression of *eya* and *so* using RNAi interference (RNAi) and analyzing Cut expression as marker for cone cell development. We used *GMR-Gal4* to drive *so-RNAi* and *eya-RNAi* in all cells posterior to the MF. Knockdown of *so* or *eya* was confirmed by antibody staining (Figure S3A-D, E-H). We observe a severe reduction of Cut expression in animals with knockdown of either *eya* or *so* (Figure 5A, E), further supporting the hypothesis that *eya* may be required for cone cell development. Cut expression and eye development are normal in animals carrying the *eya-RNAi* or *so-RNAi* transgenes alone (Figure 5I-L, M-P). In addition, knockdown of *eya* or *so* does not significantly affect neuronal differentiation in the third instar eye disc as assayed by Elav staining (Figure 5B, F). However, adult eyes with *so* or *eya* knockdown have a glazed appearance, consistent with a cone cell defect (Figure 5D, 5H) [34]. To further test for a role for *eya* expression in cone cell development we examined pupal retinas of the rare *eya^ΔPSE^* animals. The pupal retinas of *eya^ΔPSE^* animals show a clear loss of cone cells (red asterisk in Figure 3B) compared to *eya^GR^* animals (Figure 3A). Additionally, some ommatidia in the *eya^ΔPSE^* retina appeared to be fused (yellow asterisk in Figure 3B). Unexpectedly, pupal retinae of *eya^ΔPSE^* animals show an excess of interommatidial cells (compare arrowheads in Figure 3A and B). Consistent with these observations, sections of *eya^ΔPSE^* adult retinas show severely disorganized ommatidia with some ommatidia showing less than the normal complement of rhabdomeres as well as increased pigment cells (Figure 3E and F).

### Loss of *eya* expression activates Hedgehog signaling posterior to the MF

Previous studies have suggested that ectopic expression of Hedgehog (Hh), as well as the Hh nuclear effector Cubitus interruptus (Ci), can cause an increase in the number of pigment cells in the *Drosophila* eye [35]. In addition, loss of *Cul3* posterior to the MF causes elevated levels of Ci and also leads to an increase in the number of pigment cells [35]. Given the elevated numbers of pigment cells in *eya^ΔPSE^* animals, we investigated if elevated levels of Hh signaling persist in retinal cells harboring a deletion of *eya-PSE*. Hh is secreted by differentiating photoreceptors posterior to the MF and activates expression of target genes via the nuclear effector Cubitus interruptus (Ci) in the MF. Retinal progenitors in the MF stabilize the active form of Ci (Ci^Act^) that activates transcription of Hh responsive genes such as *dpp*. However, Ci is actively degraded in all the cells posterior to the MF [36]. We investigated if reduced levels of *eya* expression would lead to higher levels of Ci^Act^. We analyzed expression of Ci in *eya^ΔPSE^* clones using an antibody that recognizes both the full length and activated forms of Ci [37]. We find that reduced *eya* expression leads to elevated expression of Ci both anterior as well as posterior to the MF (Figure 3J-L), suggesting that *eya* may play a role in Ci degradation both anterior and posterior to the MF.

### Regulation of *eya-IAM* and *eya-PSE* enhancers

*eya* receives regulatory input from several signaling pathways in the eye, as well as RD network members such as Ey [38], [39]. To identify potential direct regulators of *eya-IAM* and *eya-PSE*, we mutated putative binding sites of potential regulators within these enhancers, including Ey and Mad binding sites in *eya-IAM*, as well as So and several putative ETS binding sites in *eya-PSE*. Mad is the downstream effector of the Dpp pathway, which has been shown to activate Eya expression in the eye [9], and the ETS family transcription factor Pointed acts downstream of the Egfr pathway in differentiating eye cells [39]. None of these mutations caused any noticeable changes in enhancer activity (data not shown). Additionally, we tested if *eya-IAM* expression was altered in *Mad^1-2^, smo^3^* double mutant clones that cannot respond to Dpp or Hh signaling in order to rule out indirect regulation by Dpp and Hh pathways. Consistent with the mutant binding site data, *eya-IAM* expression was unaltered in the *Mad^1-2^, smo^3^* double mutant clones.

**Figure 6:**
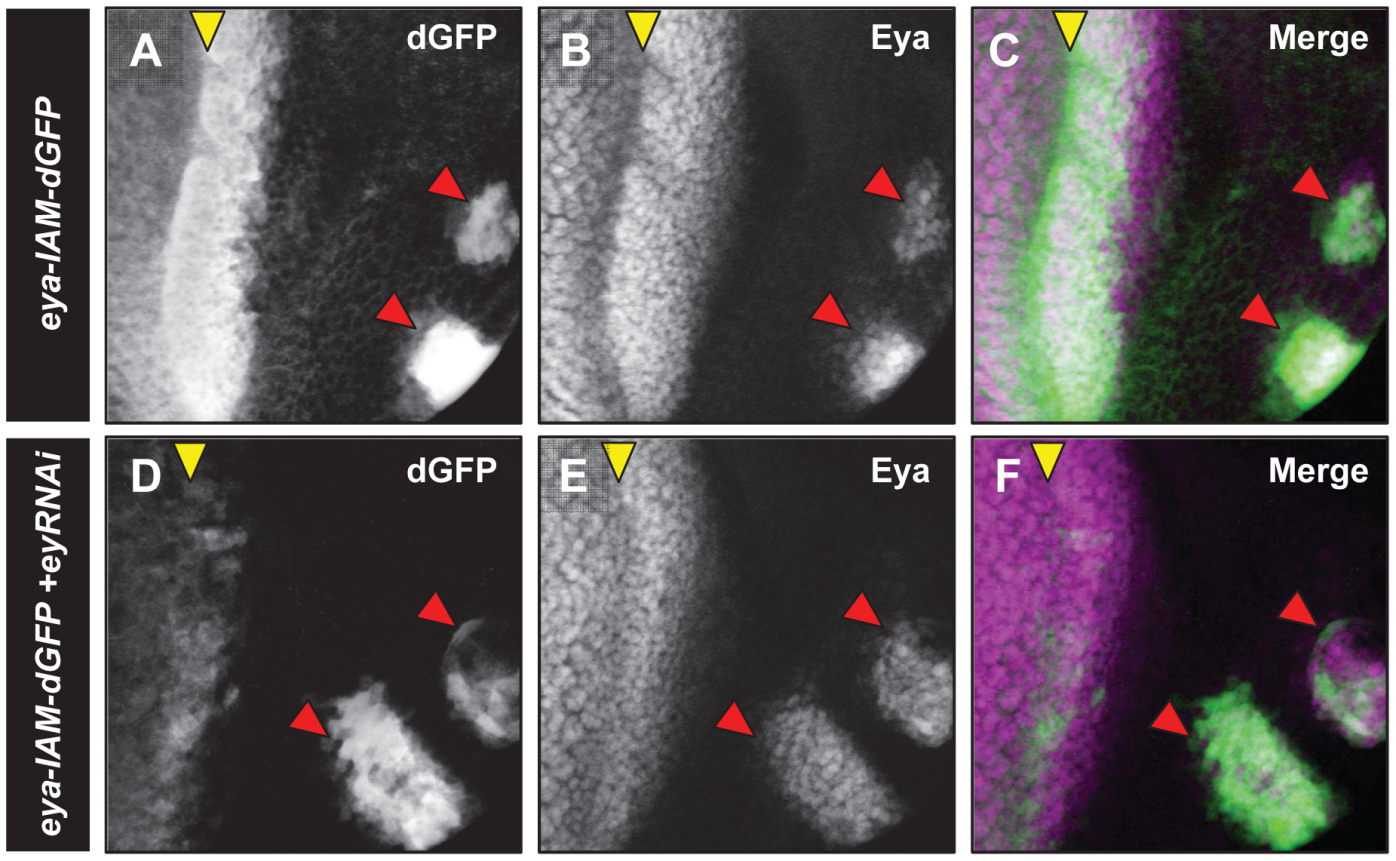
*eya-IAM-dGFP* expression is regulated by *ey* immediately anterior to the MF. Third instar eye discs showing dGFP expression (white in A and D, green in C and E) and endogenous Eya expression (white in B and E, magenta in C and E) in *hairy-Gal4*; *eya-IAM-dGFP* (A-C) and *hairy-Gal4*/*ey-RNAi; eya-IAM-dGFP* (D-F). Red arrowheads mark the position of developing ocelli.

Mutation of a putative Ey binding site [40] did not alter *eya-IAM* driven dGFP expression. However previous reports show loss of Eya expression in *ey* mutant eye discs [41]. Therefore, it is possible that *eya-IAM* is regulated by Ey through a non-canonical site or indirectly via other transcription factors. To this end, we reduced levels of Ey anterior to the MF using *ey-RNAi* driven by *hairy^h10^-Gal4* [42]. We find that in *ey* knockdown animals (*ey-RNAi/eya-IAM; hairy^h10^-Gal4*/+), dGFP expression anterior to the MF is markedly reduced (Figure 6). These results suggest that Ey regulates *eya-IAM* anterior to the MF, either directly or indirectly, through an unknown binding site.

## Discussion

### New tools to analyze the role of *eya* during retinal development

Many of the developmental events that take place anterior to the MF are dependent on each other for proper coordination and timing. For example, initiation of retinal specification marked by *ato* expression cannot take place without G1 arrest of retinal progenitors and G1 arrest in turn depends on the signaling from the MF. Loss of *eya* function in the developing eye disc blocks MF initiation and the remaining anterior retinal cells continue to proliferate and express *ey* during the early third instar stage [10], . Therefore, previously existing tools, such as clonal analysis using strong loss-of-function *eya* alleles, are not ideally suited to decipher the roles of *eya* in the plethora of developmental events that take place in retinal progenitors anterior to the MF or in differentiating cells posterior to the MF.

In this report we have identified two new *cis* enhancers of *eya* that are needed to regulate *eya* expression during eye development. Deletion of either the *eya-IAM* or *eya-PSE* enhancer leads to a reduction in the eye field or a glazed eye phenotype, respectively. *eya^ΔIAM^* and *eya^ΔPSE^* clones show significantly reduced levels of *eya* expression; together, these observations suggest that loss of these enhancers reduce *eya* expression while still allowing initiation of retinal differentiation. Thus, *eya^ΔIAM^* and *eya^ΔPSE^* clones have a distinct advantage over *eya^cliIID^* null clones in that they retain some ability to undergo retinal morphogenesis and differentiation. Therefore, *eya^ΔIAM^* and *eya^ΔPSE^* clones can be used to dissect developmental networks that are sensitive to the levels of Eya during retinal development, both anterior as well as posterior to the MF.

### *eya* is required anterior to the MF to regulate G1 arrest and Ato expression

Gain- and loss-of-function studies have supported a role for *eya* in developmental processes anterior to the MF, including mediating G1 arrest of retinal progenitors [16]. The *eya^ΔIAM^* clones reported here represent a more refined system to study the role of *eya* in G1 arrest. Specifically, *eya^ΔIAM^* clones show reduced levels of *eya* but do not cause the complete block in retinal development that is associated with the *eya^cliIID^* null mutant clones. Our analysis suggests that reduced levels of Eya lead to delayed G1 arrest as evidenced by sustained Cyclin B expression in *eya^ΔIAM^* clones adjacent to the MF. This observation is consistent with previous studies that propose a role of *eya* in mediating the G1 arrest of retinal progenitors anterior to the MF [16].

Based on its physical interaction with the transcription factor So, Eya has been implicated in regulating *ato* expression. However, this role has never been directly tested.

Our analysis of the *ato* expression in *eya^ΔIAM^* clones shows a reduction of Ato in retinal progenitors with reduced *eya* expression. These results further support an important role for *eya* in mediating G1 arrest anterior to the MF and activating Ato expression during normal retinal development.

### Cut regulation by Eya and So

Our results suggest that both *eya* and *so* are required for Cut expression in cone cells of the developing retina. This result differs from the recently published role of So-Eya in blocking Cut expression during specification of the antennal field in the second instar stage [43]. These seemingly opposite roles for So-Eya complex in Cut regulation are reminiscent of the recently reported ability of So to act both as a transcriptional activator of *eyeless* anterior to the MF as well as a transcriptional repressor of *eyeless* posterior to the MF [17]. Moreover, it is not surprising that the So-Eya complex may regulate certain genes in distinct ways during antennal specification versus cone cell development.

While the exact mechanism by which the So-Eya complex can activate or repress Cut expression is not known, our So ChIP-seq analysis does not show enrichment for So-binding at the *cut* locus [44]. However, *lz* has been shown to be a direct target of So and loss of *lz* leads to loss of Cut expression in the third instar disc posterior to the MF [14], [45]. Therefore, in the context of cone cell specification it is possible that the So-Eya complex may regulate Cut indirectly through direct regulation of *lz*.

### Role of *eya* in downregulation of Ci^Act^ levels posterior to the MF

In absence of the Hh signal, Ci - the nuclear effector of the Hh pathway - is proteolytically cleaved to a transcriptional repressor form called Ci^Rep^. In the presence of the Hh signal this cleavage is blocked and leads to stabilization of the full length transcriptional activator form called Ci^Act^. In the third instar eye disc, mature R8 photoreceptors posterior to the MF secrete Hh which diffuses to the MF and activates the Dpp signaling pathway. Thus, the full length form of Ci should be expressed in all cells posterior to the MF and within the MF. However, expression of Ci^Act^ is detected only in cells within the MF. It has been suggested that posterior to the MF, full length Ci is rapidly degraded by the Cul 3-Rdx complex [35], [36], [46]. Cul3 is a member of *Drosophila* Cullin family involved in protein degradation and Rdx is an adaptor protein encoded by *roadkill* (*rdx*). Loss of *rdx* function results in elevated expression of Ci^Act^ posterior to the MF [35], [36], [46], suggesting a critical role for *rdx* in the degradation of Ci^Act^. An *rdx-lacZ* reporter suggests that *rdx* may be expressed in all cells posterior to the MF [47]; however, the molecular mechanism regulating *rdx* expression is not completely understood. It has been suggested that the EGFR pathway may play a role in regulation of *rdx*, although whether this activation is direct or indirect is not known [47]. Our results raise the possibility that *eya* expression maybe necessary for degradation of Ci^Act^. Given that *eya* expression posterior to the MF is regulated by the EGFR pathway [39] and that *rdx* is expressed in the same cell that express *eya*, it is possible that Eya could mediate degradation of Ci via *rdx* activation. Consistent with this hypothesis, we found that *rdx* is enriched for So-binding (the binding partner of Eya) sites in third instar eye discs [44]. Thus, our observations, together with previous reports, raise the possibility that the So-Eya complex may be necessary for timely degradation of Ci^Act^ posterior to the MF. Previous work from our laboratory has shown that the proteolytically cleaved form of Ci – Ci^Rep^ blocks activation of *eya* expression [48]. Thus a complex interplay between Eya and Hh may be required for precise regulation of eye development.

### Mechanism for *eya* regulation of cone and pigment cell development

Our analysis of the rare *eya^ΔPSE^* escapers and the *eya^ΔPSE^* clones suggests a role for *eya* expression in cone and pigment cell development. Previous reports suggest that upregulation of Ci posterior to the MF can lead to excess pigment cells [35], [36], [46]. The upregulation of Ci in *eya^ΔPSE^* clones suggests that elevated Ci levels may contribute to excess pigment cells. However, reduced levels of *eya* posterior to the MF also cause defects in cone cell development. Signals from cone cells play an important role in specification of the primary pigment cell, thus raising the possibility that loss of cone cells may contribute to defective pigment cell development [3]. The tools generated in this study will be valuable in future studies of the mechanism by which *eya* regulates cone and pigment cell development.

In summary, we have identified two new *cis* regulatory regions of *eya* that are needed to maintain *eya* expression immediately anterior and posterior to the MF. We have shown that these enhancers are necessary and sufficient for proper regulation of *eya* expression during retinal development. Clonal analysis using *eya* genomic rescue constructs bearing deletions of these enhancers has shown for the first time that loss of *eya* results in a delay in G1 arrest of retinal progenitors anterior to the MF and a delay in the expression of the proneural protein *ato*. In addition, our results identify a potential role for *eya* in cone and pigment cell differentiation and timely degradation of Ci posterior to the MF.

## Supporting Information

Figure S1 **A-C**. dGFP expression in larval eye discs driven by the 322 bp enhancer at 60 hr and 74 hr AEL (A and C) and the *eya-IAM* enhancer in a 60 hr eye disc (B). Eye discs were stained for dGFP (white in A and B, green in C) and endogenous Eya (red in C). **D-E** Ocelli of *eya^cliIID^/CyO*; *eya^GRΔIAM^* (C) and *eya^cliIID^/Df(eya)*; *eya^GRΔIAM^* (D) animals. Yellow arrowheads indicate the positions of ocelli. **F-G** Eya expression in third instar eye discs (white in E and F) in *eya^2^/CyO*; *eya^GRΔIAM^* (E), and *eya^2^/Df(eya)*; *eya^GRΔIAM^* (F). **H-J.** Adult eye phenotypes of *eya^cliIID^/CyO*; *eya^GRΔIAM^* (H), *eya^cliIID^/CyO*; *eya^GRΔPSE^* (I) and *eya^2^/Df(eya)*; *eya^GRΔIAM^* (J) animals.
(TIF)

Figure S2 ***F4-dGFP*** **completely recapitulates the endogenous Eya expression pattern in mid-late third instar eye imaginal discs (A-A′″).** dGFP expression (white in A and green in A''') completely overlaps with the endogenous Eya expression (white in A′ and magenta in A''') and the neuronal differentiation marker Elav (white in A″ and red in A'''). Overlap of all three markers is shown in A'''. The *F4-dGFP* eye disc shown here is the same as the one shown in Figure 1B. Elav (white in B″) expression in *eya^ΔPSE^* clone is not significantly altered. Eya expression is reduced (white in B′). Merge is shown in B′″. The clones shown here are the same as those shown in Figure 2G-I. **C-F. The** *eya-PSE* enhancer is active in differentiating photoreceptors posterior to the MF. Top panels (C-F) show third instar eye discs from transgenic larva carrying *eya-PSE*-dGFP. Eye discs were stained for dGFP (C and C′), Elav (D and D′), and Actin (E and E′). The bottom panels show orthogonal views of the same disc. The dotted yellow line in panel D indicates the position of the orthogonal sections.
(TIF)

Figure S3 **A-H.** RNAi knockdown of *so* and *eya*. Third instar eye discs of *soRNAi/GMR-Gal4* (A-D) and *eya-RNAi*/*/GMR-Gal4* (E-H) stained for So (A and F), Eya (B and E), and Elav (C and G). Although So and Eya expression are clearly reduced in response to their respective RNAi constructs, no obvious change in ELAV expression is observed in either case.
(TIF)
