# Supplementary figures and images for: Drosophila Eyes Absent Is Required for Normal Cone and Pigment Cell Development
Source: PLoS One. 2014 Jul 24;9(7):e102143. doi: 10.1371/journal.pone.0102143 (PMC4109927; doi:10.1371/journal.pone.0102143)

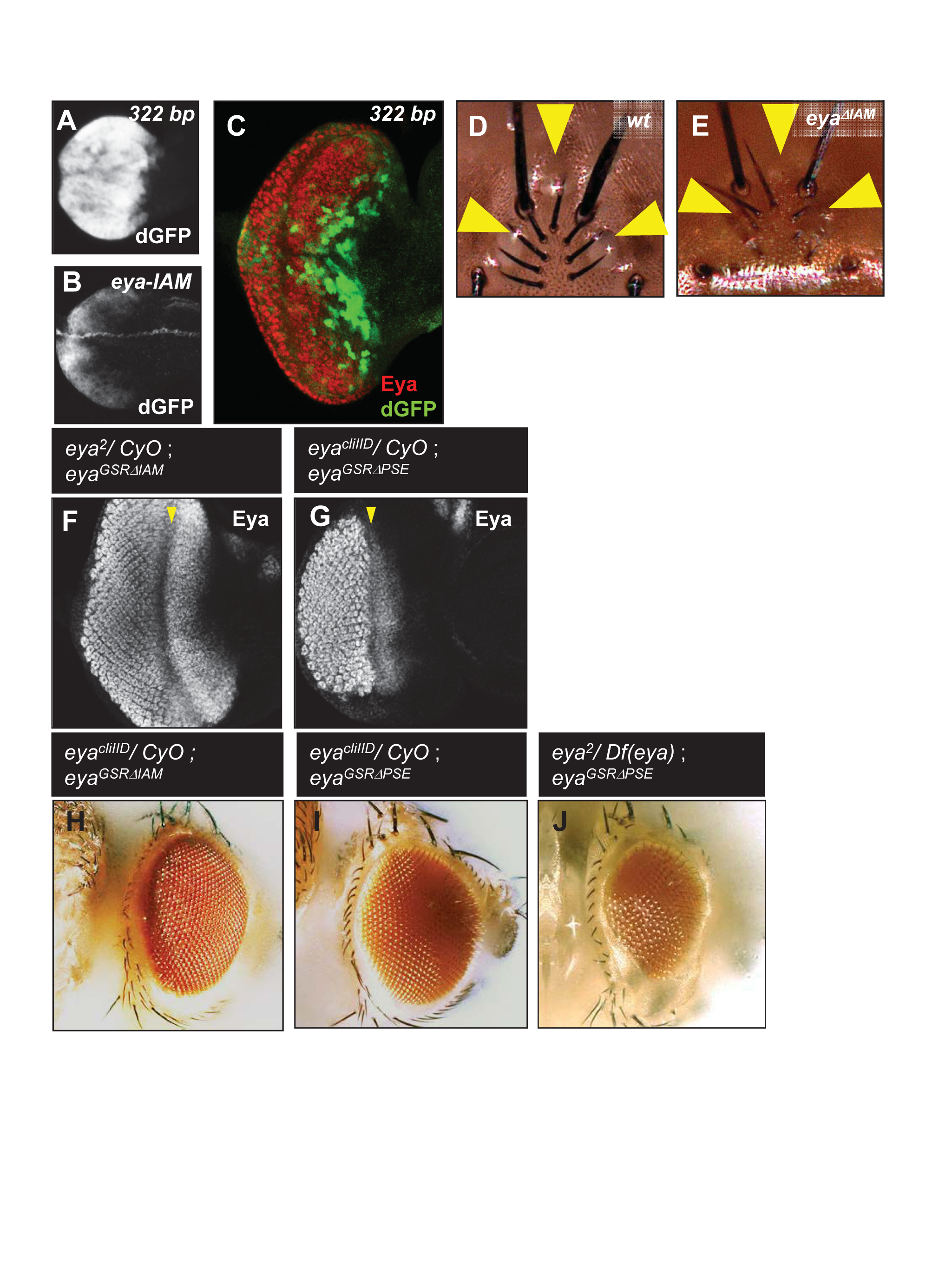

Supplement: Figure S1 — A-C. dGFP expression in larval eye discs driven by the 322 bp enhancer at 60 hr and 74 hr AEL (A and C) and the eya-IAM enhancer in a 60 hr eye disc (B). Eye discs were stained for dGFP (white in A and B, green in C) and endogenous Eya (red in C). D-E Ocelli of eyacliIID/CyO; eyaGRΔIAM (C) and eyacliIID/Df(eya); eyaGRΔIAM (D) animals. Yellow arrowheads indicate the positions of ocelli. F-G Eya expression in third instar eye discs (white in E and F) in eya2/CyO; eyaGRΔIAM (E), and eya2/Df(eya); eyaGRΔIAM (F). H-J. Adult eye phenotypes of eyacliIID/CyO; eyaGRΔIAM (H), eyacliIID/CyO; eyaGRΔPSE (I) and eya2/Df(eya); eyaGRΔIAM (J) animals. (TIF) [file pone.0102143.s001.tif]

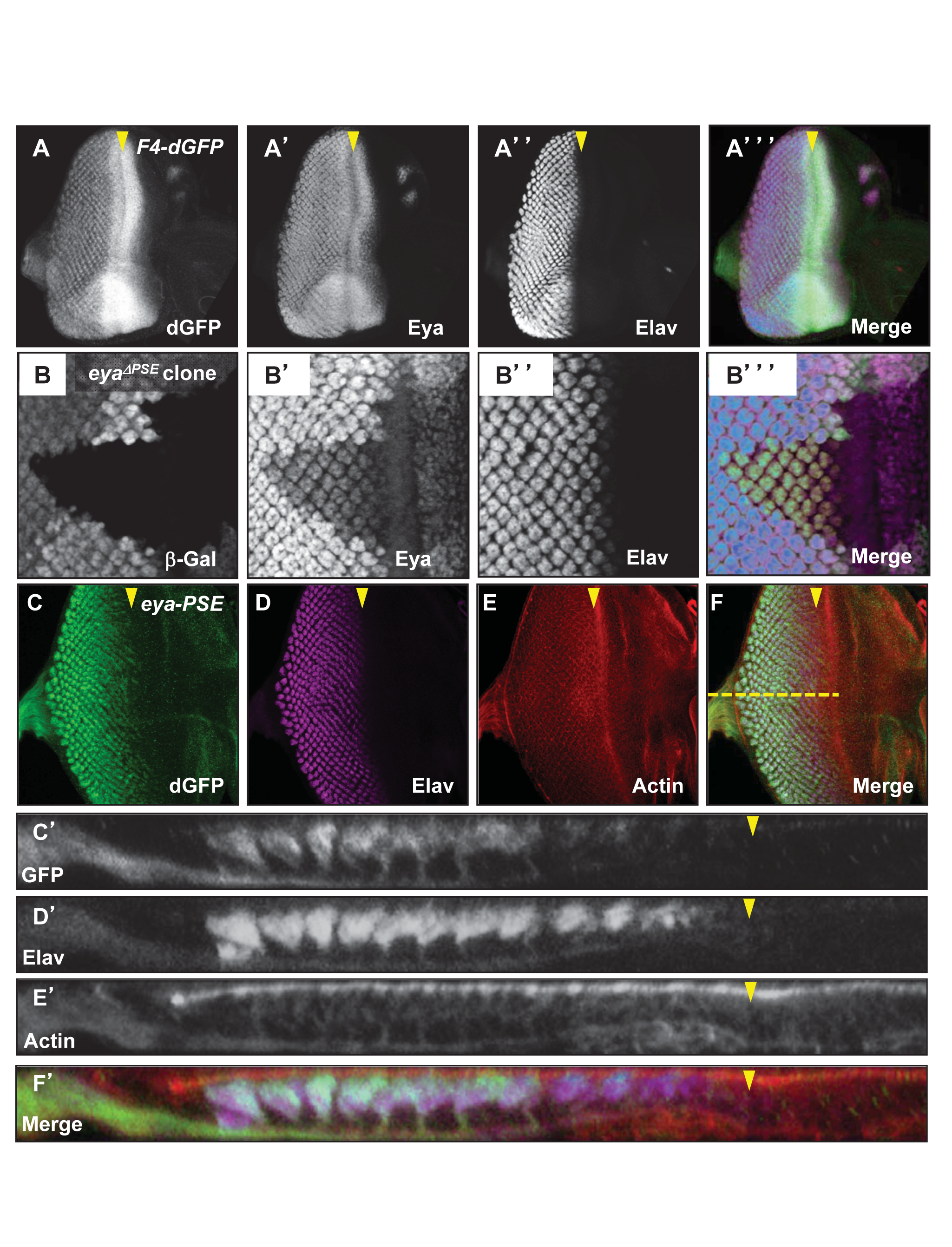

Supplement: Figure S2 — F4-dGFP completely recapitulates the endogenous Eya expression pattern in mid-late third instar eye imaginal discs (A-A′″). dGFP expression (white in A and green in A''') completely overlaps with the endogenous Eya expression (white in A′ and magenta in A''') and the neuronal differentiation marker Elav (white in A″ and red in A'''). Overlap of all three markers is shown in A'''. The F4-dGFP eye disc shown here is the same as the one shown in Figure 1B. Elav (white in B″) expression in eyaΔPSE clone is not significantly altered. Eya expression is reduced (white in B′). Merge is shown in B′″. The clones shown here are the same as those shown in Figure 2G-I. C-F. The eya-PSE enhancer is active in differentiating photoreceptors posterior to the MF. Top panels (C-F) show third instar eye discs from transgenic larva carrying eya-PSE-dGFP. Eye discs were stained for dGFP (C and C′), Elav (D and D′), and Actin (E and E′). The bottom panels show orthogonal views of the same disc. The dotted yellow line in panel D indicates the position of the orthogonal sections. (TIF) [file pone.0102143.s002.tif]

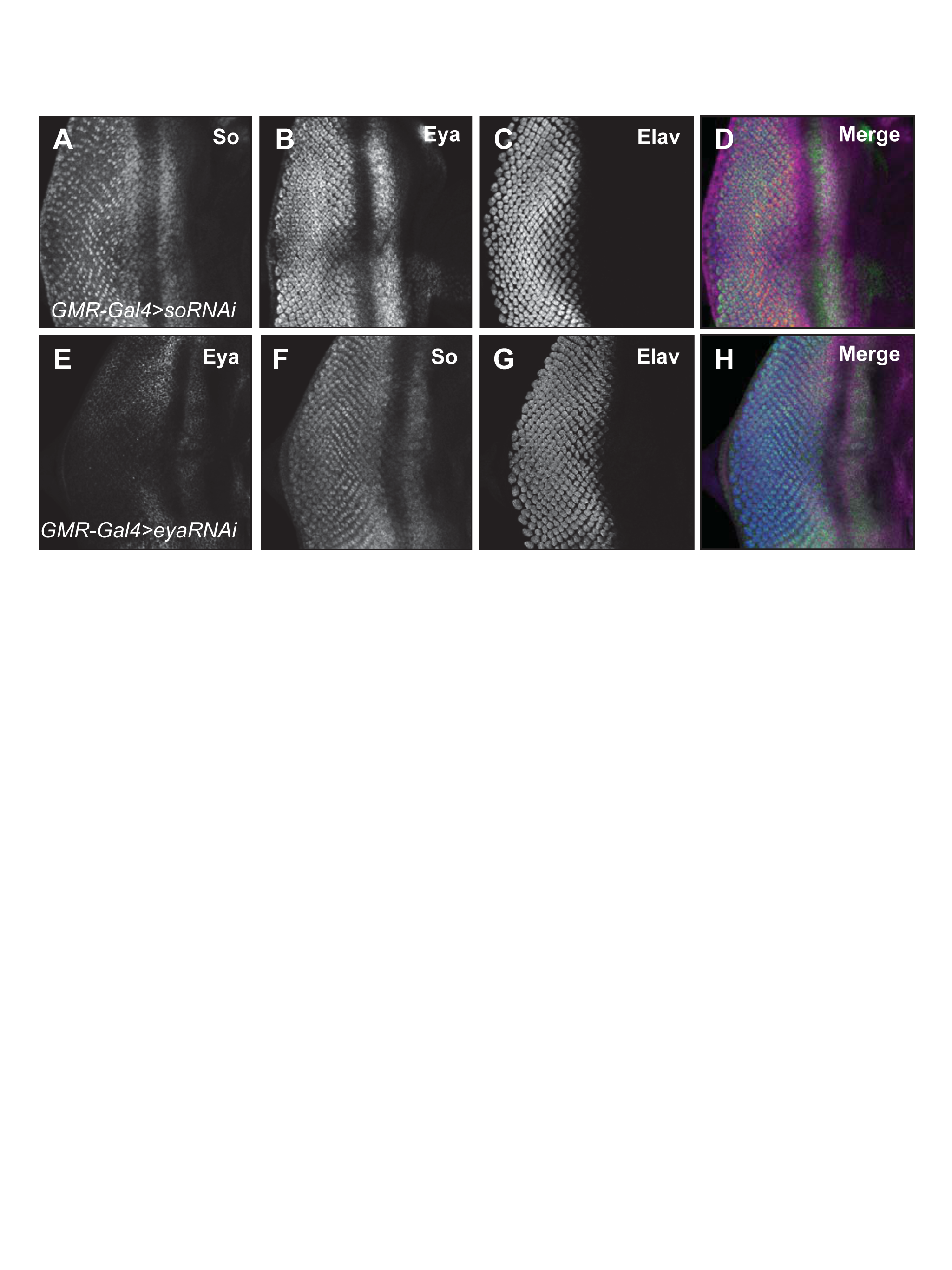

Supplement: Figure S3 — A-H. RNAi knockdown of so and eya. Third instar eye discs of soRNAi/GMR-Gal4 (A-D) and eya-RNAi//GMR-Gal4 (E-H) stained for So (A and F), Eya (B and E), and Elav (C and G). Although So and Eya expression are clearly reduced in response to their respective RNAi constructs, no obvious change in ELAV expression is observed in either case. (TIF) [file pone.0102143.s003.tif]
